# Tyrosine phosphatase activity is restricted by basic charge substituting mutation of substrates

**DOI:** 10.1038/s41598-022-19133-4

**Published:** 2022-09-05

**Authors:** Che-Fan Huang, Cara J. Gottardi, Milan Mrksich

**Affiliations:** 1 Department of Chemistry, Northwestern University, Evanston, IL 60208 USA; 2 Division of Pulmonary and Critical Care, Department of Medicine, Northwestern University, Chicago, IL 60611 USA; 3 Biochemistry and Molecular Genetics, Northwestern University, Chicago, IL 60611 USA; 4 Department of Biomedical Engineering, Northwestern University, Evanston, IL 60208 USA; 5 Department of Cell & Developmental Biology, Northwestern University, Chicago, IL 60611 USA

**Keywords:** Cell biology, Chemical biology

## Abstract

Phosphorylation controls important cellular signals and its dysregulation leads to disease. While most phospho-regulation studies are focused on kinases, phosphatases are comparatively overlooked. Combining peptide arrays with SAMDI mass spectrometry, we show that tyrosine phosphatase activity is restricted by basic amino acids adjacent to phosphotyrosines. We validate this model using two β-catenin mutants associated with cancer (T653R/K) and a mouse model for intellectual disability (T653K). These mutants introduce a basic residue next to Y654, an established phosphorylation site where modification shifts β-catenin from cell–cell adhesions and towards its essential nuclear role as Wnt-signaling effector. We show that T653-basic mutant β-catenins are less efficiently dephosphorylated by phosphatases, leading to sustained Y654 phosphorylation and elevated Wnt signals, similar to those observed for Y654E phospho-mimic mutant mice. This model rationalizes how basic mutations proximal to phosphotyrosines can restrict counter-regulation by phosphatases, providing new mechanismistic and treatment insights for 6000+ potentially relevant cancer mutations.

## Introduction

While phosphoproteome levels are determined by competing kinase and phosphatase activities, most studies address the role of kinases and assume that phosphatases play non-regulatory roles in signaling^1^. However, the number of protein tyrosine kinases (PTK) and protein tyrosine phosphatases (PTP) in the human proteome are of the same order (90 PTKs and 107 PTPs), suggesting comparable roles for the two families^2,3^. The common view that phosphatases largely play a “housekeeper” role may lack justification and is being challenged^4–7^. For example, SHP2 was identified as the first oncogenic phosphatase, demonstrating regulatory roles for PTPs, and linking dysregulated PTP activity directly to disease^8–10^. Another example found that PTP1B contributes to the development of insulin resistance and type 2 diabetes^11^.

**Figure 1:**
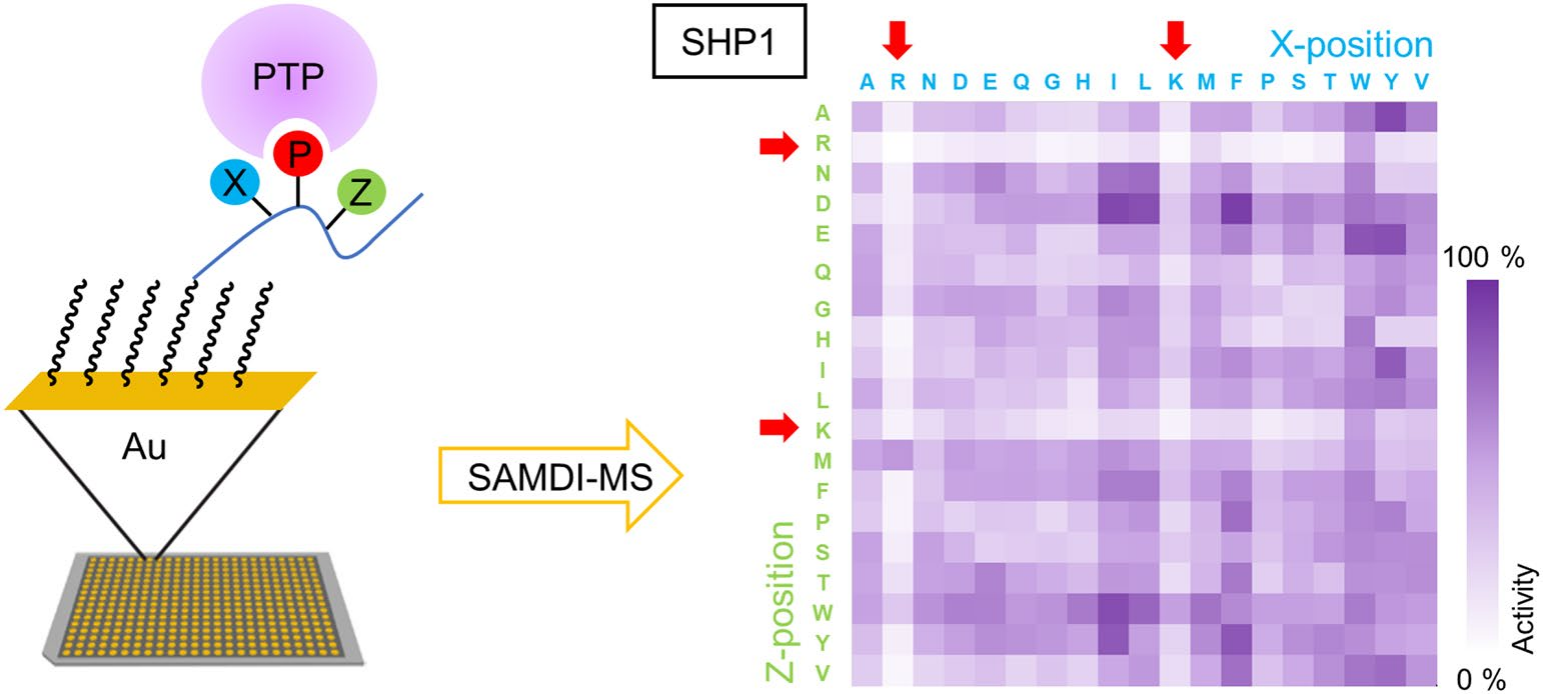
Phosphatase activity profiling using peptide arrays and SAMDI mass spectrometry. (Left) Peptides immobilized on a self-assembled monolayer against a background of tri(ethylene glycol) groups. The array is treated with PTP and dephosphorylation of each peptide is then analyzed with SAMDI-MS. (Right) Heatmap for SHP1 shows basic residues adjacent to phosphotyrosine inhibit enzymatic activity.

**Figure 2:**
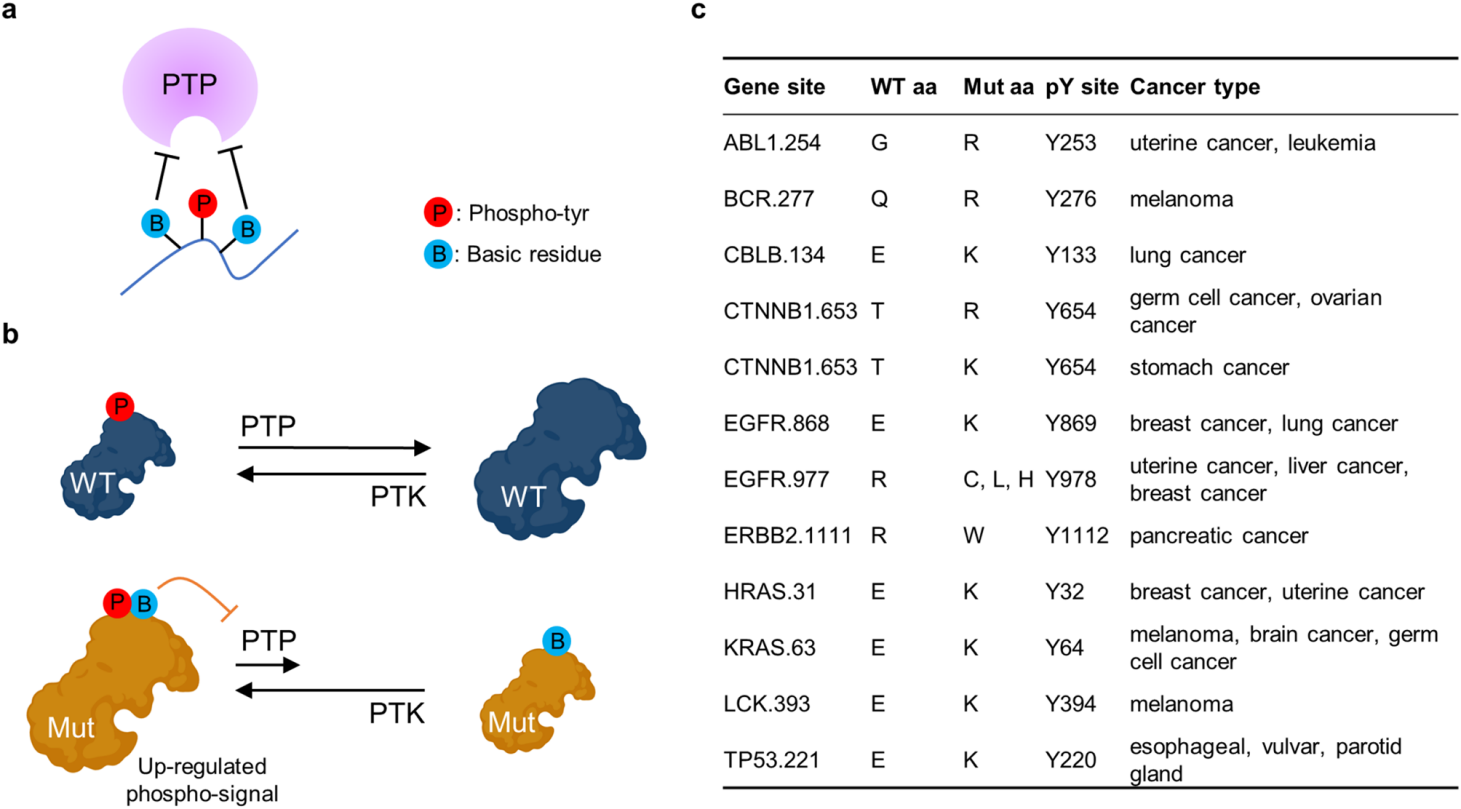
Basic residues antagonize PTP activity. (**a**) Basic residues lower the affinity of phosphotyrosine substrates for the positively-charged pocket of the PTP. (**b**) Charge-mutation/PTP-restriction mechanism: mutant with a basic residue adjacent to the phosphotyrosine antagonizes dephosphorylation, leading to persistent phospho-signal compared to wild-type. (**c**) Basic residue-substitution missense mutations identified for select cancer-relevant genes.

A broader understanding of the specificities of PTPs, along with mechanisms that regulate their activities is important to framing hypothesis to understand their roles in signaling^12–14^. The challenges associated with biochemical assays of phosphatase activity have limited progress in this direction. In recent work, we used peptide arrays and SAMDI-MS^15–21^ (self-assembled monolayers for matrix-assisted laser desorption/ionization mass spectrometry) to profile the specificities of 22 phosphatases including PTP1B, TC-PTP, MEG1, SHP1, PTPN7, MEG2, SHP2, PTPN12, PTPN13, PTPN14, PTPRA, PTPbeta, CD45, PTPRE, RPTPG, DEP1, PTPmu, PTPsigma, PRL1-3 and hALP (Fig. 1)^22^. We found that most of the PTPs lack activity towards substrates that have an arginine (R) or lysine (K) residue adjacent to the phosphotyrosine (pY). The results agree with previous observations that PTPs favor acidic residues^23^ and the PTP selectivity against basic residues was also described in earlier reports by Pei et al.^24–26^. In subsequent work, we used peptide arrays to profile the PTP activity in 5 different cell lysates, and in each case we found that the global activity disfavored substrates having a basic residue adjacent to the pY site (Fig. 2a)^27^.

These studies suggest the possibility that charge-substitution mutations proximal to pY sites could impact a range of signaling pathways and disease pathology. Here we address the hypothesis that basic amino acid missense mutations adjacent to phosphotyrosine residues restrict PTP-mediated dephosphorylation (Fig. 2b). Through a database search, we identified 6000+ cancer mutations where basic residues are added or removed adjacent to the phosphotyrosine sites^28,29^. We identified β-catenin (β-cat) as a relevant example, because it has a T653K mutation next to the pY residue, and is associated with intellectual disability and syndromic features (batface, *Bfc*) in mice^30^; this and the analogous T653R mutation are also found in the cancer genome^31^.

We reasoned that the higher phosphorylation level of Y654 in the mutants is due to decreased phosphatase activity, and which would lead to elevated Wnt signals and associated disease. Below, we demonstrate that introduction of a basic residue adjacent to Y654 in β-cat antagonizes its dephosphorylation by SHP1 in HEK cells, favoring persistent phosphorylation and elevation of AXIN2, which is a canonical β-cat target in the Wnt pathway^32^. This mechanism rationalizes neuro-developmental phenotypic similarities between T653K *Bfc* and Y654E phospho-mimic mutant mice^33^, as well as how β-cat missense mutations proximal to Y654 may drive epithelial cancers.

## Results

### Database search identifies 6000+ relevant cancer mutants

To identify cancer mutants with basic residue substitutions adjacent to phosphotyrosines, we employed the protein post-translational modification (PTM) database PhosphoSitePlus and the single-nucleotide variations (SNV) cancer database BioMuta^28,29^. We used PhosphoSitePlus to identify all reported phosphotyrosine sites and selected for those that had corresponding cancer mutations at their + 1 and − 1 positions in BioMuta. We then filtered these sites to select those with a R or K amino acid in either the wildtype or mutant forms, to identify base-substitution missense cancer mutations including both addition and removal of basic residues. Some notable sites identified are listed in Fig. 2c and a comprehensive list of the database search results can be found in SI Table 1. To our surprise, this search identified more than 6000 mutants, though most had an unclear contribution to protein function. While these pY-proximal missense mutations occur frequently, how they impact phosphatase activity (as opposed to kinase activity) is poorly understood.

We selected the *CTNNB1* gene/β-cat protein for further study, because T653R and T653K are proximal to the well-studied Y654 phospho-site and are associated with reduced cell–cell adhesion and elevated nuclear signaling. Moreover, β-cat T653K was also identified in a forward genetic screen for mutations affecting craniofacial development in mice, generating phenotypes that overlap with dominant human β-cat/*CTNNB1* mutations causing intellectual disability with similar syndromic features^30^.

### Phospho-dependent signaling of β-catenin

β-cat is a canonical junction-nuclear signaling protein. As a physical link between cell surface cadherins and the actin-binding protein α-catenin, β-cat plays a critical role in cell–cell cohesion. Outside of the cadherin-catenin complex, β-cat also functions as an essential effector of the Wnt pathway, pairing with DNA-binding factors to activate transcription of genes that drive cell fate decisions^34^. The degree to which β-cat participates in these two functions is determined by the availability and relative affinity of β-cat binding with its competing partners^35^. One modification that shifts β-cat from its cell–cell adhesive function in favor of nuclear signaling is its phosphorylation at Y654 by Src family kinases (SFKs) where this modification reduces β-cat binding to the cadherin cytoplasmic domain^36,37^ and also antagonizes its degradation by the β-cat phospho-destruction complex^38,39^, leading to additional modifications that enhance transcriptional activity^33^. Below, we sought to determine whether β-cat basic T653K/R mutants lead to enhanced Wnt signaling through persistent phosphorylation of Y654 due to lack of PTP activity.

### β-cat T653 basic mutants show greater phosphorylation at Y654

**Figure 3:**
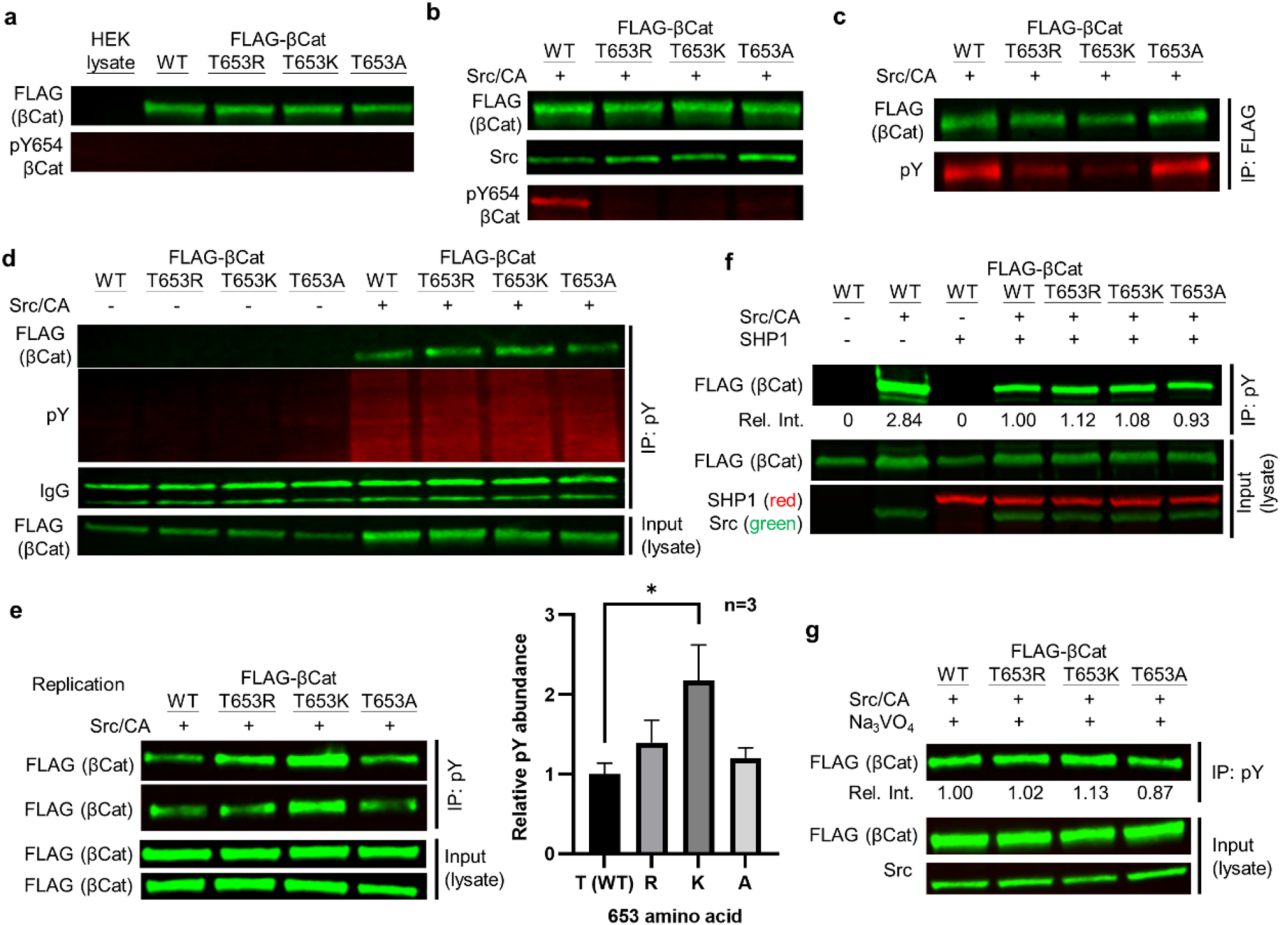
β-cat phosphorylation at Y654 is enhanced by basic amino acid replacement at T653. (**a**) Four β-cat constructs were transfected and expressed equally in HEK 293 T cells. (**b**) Co-transfection of Src/CA promotes β-cat Y654 phosphorylation. Site-specific antibody does not recognize mutants. (**c**) Equal amount of lysates (1 mg) were immunoprecipitated with a FLAG antibody. Pan-phosphotyrosine antibody displays different binding affinity to WT and mutants.(**d**) β-cats were immunoprecipitated with pY and blotted for FLAG to quantify their Y654 phosphorylation. (**e**) pY654 is quantified by first taking the ratio of FLAG signal in pY-pulldown and the lysate input to account for loading/expression differences. Then, the corrected intensities of mutants were compared to WT. In biological triplicate experiments, both basic β-cat T653 mutants are more phosphorylated than WT. Error bars: one standard deviation. *: p < 0.05 in t-test. (**f**) β-cat pY654 is a substrate for SHP1. (**g**) Inhibiting PTPs with pervanadate (100 μM) eliminates the difference in Y654 phosphorylation between WT and mutants.

We obtained a FLAG-β-cat /pcDNA3 plasmid^40^ and generated two basic mutants T653R, T653K and the neutral mutant T653A as control. All four constructs expressed similarly in transfected HEK 293T cells. Consistent with previous studies^39^, no Y654 phosphorylation was observed in these cells using an anti-β-cat pY654 site-specific phosphoantibody (Fig. 3a). Co-transfection with constitutively active Src kinase (Src/CA) led to robust detection of WT β-cat with the β-cat-pY654 specific antibody (Fig. 3b). Unfortunately, this antibody was unable to distinguish phosphorylation of our mutant β-cats where the adjacent T653-residue was altered, because the epitope includes adjacent residues (A-**T**-pY-A-A). We next repeated this experiment with a general phospho-tyrosine antibody (clone PY20). We found, however, that even a pan-pY antibody recognized the WT and T653A mutant β-cat proteins better than the T653K/R basic mutants (Fig. 3c), consistent with evidence that the PY20 antibody also disfavors substrates with basic residues adjacent to pY^41^. Thus, even the pan-phosphotyrosine antibody, PY20 will underreport the pY abundance for these protein sequences.

While the pan-PY20 antibody fails to recognize all pY sequences equally, it does have sufficient affinity for each of the β-cat constructs. Hence, we expected that an excess of the PY20 antibody could quantitatively immunoprecipitate the four phosphorylated constructs (including exogenously expressed FLAG-tagged β-cats). Subsequent immunoblotting for the FLAG epitope then gives β-cat pY status (Fig. 3d). With this approach, we found that the β-cat T653K mutant showed significantly (p = 0.0120, t-test) more phosphorylation by Src/CA than did WT; T653R mutant also exhibited slightly more phosphorylation than did WT and T653A, consistent with our in vitro peptide library profiling results (Fig. 3e). To explore the role of phosphatase regulation of this site, we co-transfected SHP1, a known PTP to dephosphorylate β-cat pY654^39^, along with β-cat constructs and Src/CA. We found that the amount of phospho-β-cat was reduced by roughly threefold, suggesting β-cat-pY654 is a possible target of SHP1 in vivo (Fig. 3f). Importantly, treatment with the pan-tyrosine phosphatase inhibitor, sodium pervanadate (Na_3_VO_4_) eliminated differences in phosphorylation between WT and mutant β-cat proteins (Fig. 3g). This data demonstrates that β-cat with a basic residue adjacent to pY results in higher phosphorylation levels at pY654, and that inhibition of PTPs eliminates the observed differences in phosphorylation levels. These data strongly suggest that differential phosphatase activity (not necessarily SHP1) towards β-cat pY654 versus WT β-cat can explain differences in phospho-abundance, where PTP activity is restricted by basic residues adjacent to the pY site. We cannot exclude the possibility that some of the SHP1-mediated reduction in β-cat pY654 phosphorylation in cells may be indirect, for example through inhibition of Src/CA signaling via Y419^42^.

### β-cat basic charge mutants manifest enhanced Wnt-signaling

**Figure 4:**
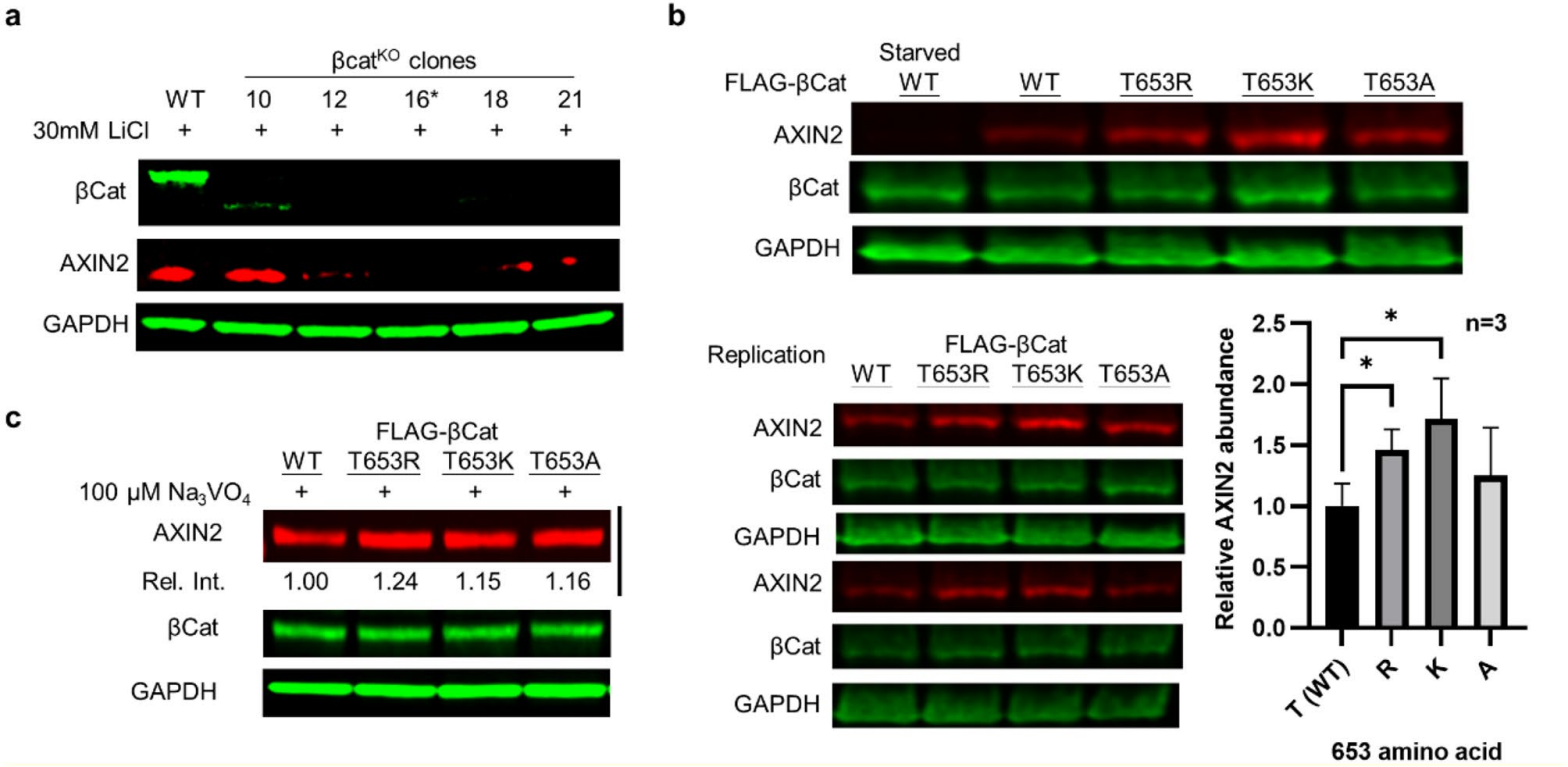
β-cat T653 basic charge mutants display enhanced Wnt-signaling. (**a**) HEK β-cat^KO^ cells generated with CRISPR-Cas9 displays a lower AXIN2 expression level treated with LiCl. Clone 16 was used for the studies. (**b**) In biological triplicate experiments, T653R and T653K β-cats induce more AXIN2 protein expression than WT β-cat. Error bars: one standard deviation. *: p < 0.05 in t-test. (**c**) PTP inhibition with pervanadate (100 μM) eliminates differences in AXIN2 levels between WT and mutant β-cats.

To gain additional evidence for increased phosphorylation levels in the β-cat T653K/R mutants, and to address how the β-cat T653K/R mutants impact Wnt-signaling, we analyzed AXIN2 levels in western blots as a downstream reporter. This protein is a universal target gene and negative-feedback regulator of the pathway^32^. We used the CRISPR-Cas9 strategy to generate a HEK β-cat^KO^ cell line (See SI for the generation and characterization of the knockout cells) and selected clones lacking AXIN2 induction upon treatment with the Wnt pathway agonist and GSK3 inhibitor, LiCl (Fig. 4a, clone 16)^32^. We transfected FLAG-tagged constructs into the β-cat^KO^ cells, serum-starved the cells overnight and then restored cultures with 5% FBS for 6 h before harvesting. We found AXIN2 was largely depleted after transfected cells were serum starved (Fig. 4b, first lane) and increased only after cells were supplied with FBS, allowing us to effectively synchronize our cells.

Using this method, we found that the T653R and T653K mutants expressed significantly more AXIN2 than WT (Fig. 4b; p = 0.0308 and 0.0301, t-test); the changes in AXIN2 expression in T653A mutant were not significant compared to WT (p = 0.3732, t-test). These findings are consistent with the elevated pY654 phosphorylation observed in Fig. 3e, and demonstrate that the more phosphorylated basic mutants contribute to elevated β-cat signaling. To show that phosphatase activity was required for the different levels of β-cat signaling, we treated cells with 100 μM Na_3_VO_4_ to inhibit PTPs and found that WT β-cat induced AXIN2 to similar levels as the T653K/R mutants (Fig. 4c). Together, these experiments establish that disease-associated missense mutant forms of β-cat (T653K/R) display enhanced Wnt-signaling activity through a positive-charge gating (or steric hindrance) mechanism that restricts tyrosine phosphatase substrate selectivity at pY654.

## Discussion

**Figure 5:**
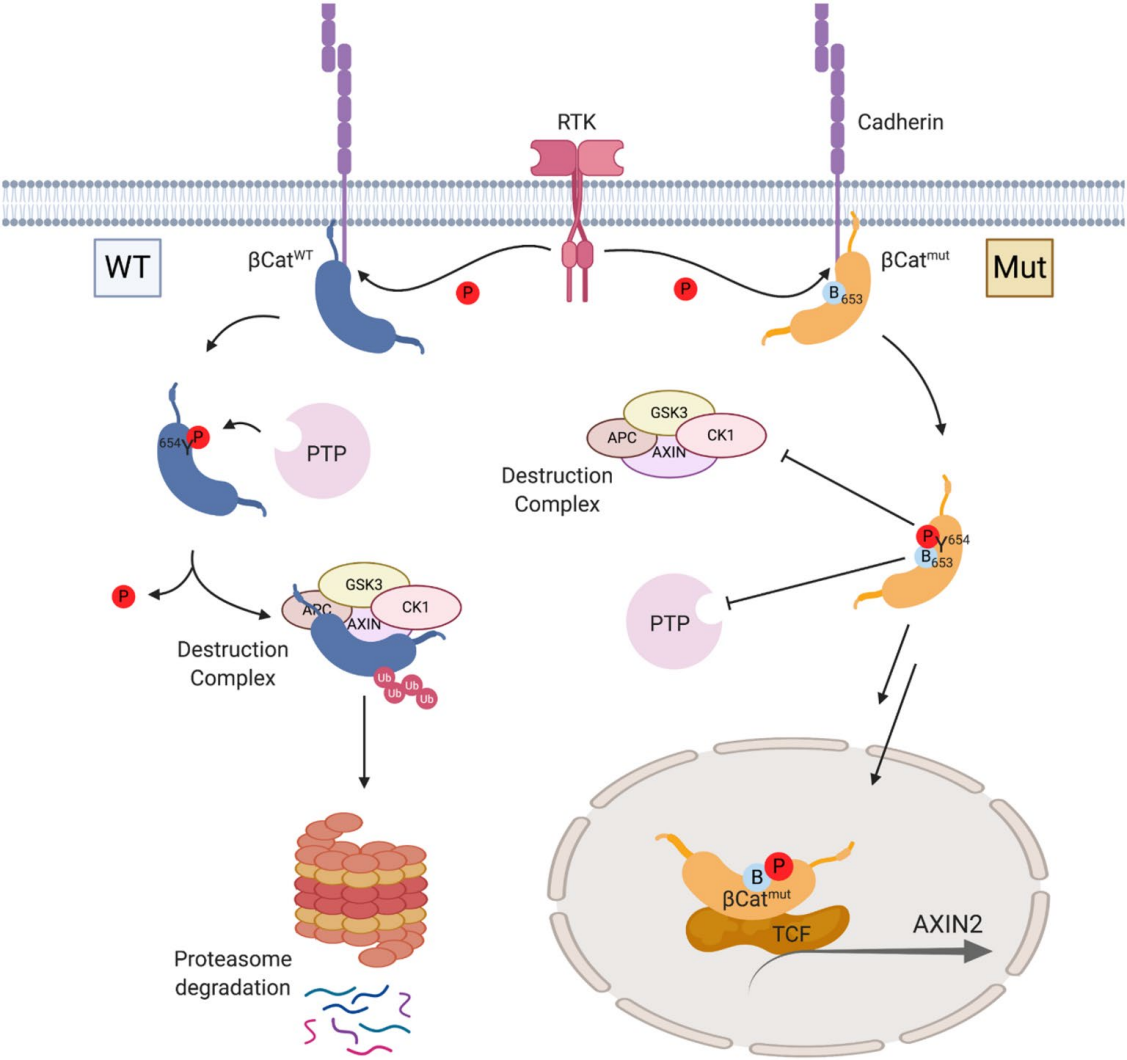
β-cat charge-mutation/PTP-restriction mechanism that enhances Wnt signaling. (Left) WT pY654 is dephosphorylated by PTPs and degraded. (Right) Basic mutants inhibit pY654 dephosphorylation. Sustained phosphorylation of β-cat reduces association with cadherins and leads to enhanced transcriptional co-activation with T-cell factor (TCF), revealed by the canonical target and negative regulator, *AXIN2*. Schematic created with Biorender.com.

This paper shows how basic residues adjacent to pY sites in proteins can antagonize dephosphorylation and result in higher phosphoprotein levels, and lead to corresponding impacts in signaling pathways. We identified β-cat as a relevant example, because T→R/K mutations are associated with disease but remain poorly understood. Our work shows that both basic mutants have higher tyrosine phosphorylation levels and lead to higher levels of AXIN2 in cells, consistent with elevated Wnt signaling compared to WT β-cat (Fig. 5). Importantly, this difference is eliminated when cells are treated with a phosphatase inhibitor, suggesting that phosphatases differentially target mutant versus WT β-cat. This work offers an alternate explanation for neuro-developmental phenotypic similarities between Y654E phospho-mimic and T653K *bfc* mutant mice^30,33^. The less efficient dephosphorylation of pY654 with an adjacent basic residue favors persistent phosphorylation and elevation of β-cat-mediated gene expression, rationalizing its resemblance to a permanent phospho-mimic in the developmental process. Our study also points to a potential SHP1 activation treatment strategy through induction of SHP1 expression or small molecule activators^43^.

Assays of phosphatase activity are very challenging, and largely not well-suited to direct determine phosphatase specificity. Traditional assays frequently use generic and non-specific small molecule substrates such as *para*-nitrophenylphosphate (*p*NPP) and 6,8-difluoro-4-methylumbelliferyl phosphate (DiFMUP), which do not provide sequence context^44,45^. One approach described by Cesareni and co-workers used peptide arrays but required the use of a substrate-trapping mutant that may not retain the substrate specificity of the wild type enzyme^14^. In a combinatorial approach using libraries of phosphopeptides immobilized to beads, Pei and co-workers identified peptides that were active substrates for the PTPs^24–26^. However, the use of a tyrosinase to label the active beads could impart its own sequence specificity and the combinatorial approaches do not provide quantitative results for every peptide sequence in the library. For example, fewer than one percent of the peptides were analyzed, giving an incomplete understanding of the enzyme specificity. SAMDI-MS is a label-free and high throughput assay that directly address these issues and provides a more complete assessment of the PTP specificity.

These benefits of the label-free assay allowed us to use SAMDI-MS to analyze peptide arrays and revealed general rules for the sequence dependent activity of phosphopeptide substrates. Remarkably, the rule we describe—where PTPs disfavor basic residues proximal to pY—is surprisingly general. In this work, we show how this insight can explain signaling in β-cat mutants and in previous work we addressed its role in insulin receptor^22^. We note that our database search identified over 6000 relevant cancer mutations that could employ this missense mutation/PTP restriction mechanism, and suggests that this mechanism can have a broad relevance in understanding mutations that underlie cancers.

This work demonstrates that disease-related mutants can operate by altering phosphoprotein levels through a modulation of phosphatase activity. The study is also significant because the underlying hypothesis was revealed through biochemical studies of PTP specificity, which was enabled by the development of a robust label-free assay. We expect that reports of mechanistic roles for phosphatase-directed phenotypes will become more common and lead to new diagnostic markers and therapeutic targets in drug discovery.

## Methods

### Database search

Phosphotyrosine sites were downloaded from PhosphoSitePlus and single-nucleotide variations (SNVs) in cancer were downloaded from BioMuta^28,29^. The cancer mutations at + 1 and − 1 positions to a phosphotyrosine that added or removed a basic residue (R or K) were extracted. A full list of identified gene sites and local sequences can be found in Table S1 and S2.

### Plasmids and site-directed mutagenesis

β-cat/pcDNA3 plasmid was a generous gift from Dr. Eric Fearon. Site-directed mutagenesis kits (New England Biolabs) were used to generate β-cat/pcDNA3 T653R, T653K and T653A mutants following the manufacturers protocol. Primers [5′-AGG TGT GGC GAG ATA TGC AGC TGC TG-3′ (R forward), 5′-AGG TGT GGC GAA ATA TGC AGC TGC TG-3′ (K forward), 5′-AGG TGT GGC GGC ATA TGC AGC TGC TG-3′ (A forward), and 5′-TCA TTC CTA GAG TGA AGT AAC TCT GTC AGA G-3′ (reverse)] were purchased from Integrated DNA Technologies. Src/CA(Y530F)/pDEST40 (#124659) and SHP1/pJ3 (#8572) plasmids were purchased from addgene. All plasmids were transformed to DH5α *E. coli.* (New England Biolabs), amplified and purified with DNA maxiprep kits (Invitrogen). All sequences were confirmed by Sanger Sequencing (ACGT, Inc.).

### Antibodies

The following primary antibodies were used in this study: mouse anti-FLAG (F3165, MilliporeSigma), rabbit anti-FLAG (F7425, MilliporeSigma), rabbit anti-pY654-β-cat (ab59430, abcam), rabbit anti-Src (701396, Invitrogen), mouse anti-phosphotyrosine (clone PY20, P4110, MilliporeSigma), mouse anti-SHP1 (MA5-11669, Invitrogen), rabbit anti-β-cat (clone RM276, SAB5600086, MilliporeSigma), mouse anti-β-cat (610153, BD Bioscience), mouse anti-γ-catenin (610253, BD Bioscience), rabbit anti-AXIN2 (PA5-25331, Invitrogen), mouse anti-GAPDH (CB1001, MilliporeSigma).

### Cell culture

HEK 293 T cells were obtained from American Type Culture Collection (ATCC) and maintained in Dulbecco’s Modified Eagle’s Medium (DMEM, Corning), containing 10% fetal bovine serum (FBS, Atlanta Biologicals), 100 U/ml penicillin and 100 mg/mL streptomycin (Corning) at 37 °C and 5% CO_2_.

### Transfection and cell lysis

For one reaction, plasmids (1 μg each) and Lipofectamine 2000 (3 μL, Invitrogen) were diluted in 125 μL Opti-MEM (Corning) respectively and mixed. The mixture was incubated for 30 min at room temperature. HEK cells were plated in 6 well-plates at a seeding density of 1.0 × 10^6^ cells in 1.25 mL Opti-MEM. The reaction mixture was added to the cells and incubated for 3 h at 37 °C and 5% CO_2_. DMEM completed with FBS and antibiotics (1.5 mL) were added. The cells were incubated for two day, washed with PBS and harvested in cell lysis buffer [50 mM Tris, 150 mM NaCl, 5 mM EDTA, 1% Triton X100, pH 7.5, cOmplete™ Protease Inhibitor (1 tablet/10 mL, Roche), PhosSTOP™ (1 tablet/10 mL, Roche), 200 uM Na_3_VO_4_.]. Lysates were sonicated and centrifuged at 14,000 rpm for 10 min to remove debris. The concentrations were determined by Bradford assay (5000006, Bio-Rad). For AXIN2 immunoblotting, the transfected cells were serum-starved overnight and incubated with DMEM complete for 6 h before lysis.

### Immunoprecipitation

Primary antibodies (10 μg) were added to 1 mg cell lysate diluted to 0.5 mL with cell lysis buffer and incubated overnight at 4 °C under agitation. Protein A-agarose beads (~ 50 μL, MilliporeSigma) were added and incubated for 1 h at 4 °C under agitation. Immunocomplexes were collected by centrifugation and washed four times with ice-cold cell lysis buffer. Proteins were solubilized with 30 μL 3 × SDS loading buffer (150 mM Tris, 4.5% SDS, 240 mM DTT, 0.12% Orange G, 30% glycerol, pH 7.5) and separated by SDS-PAGE.

### Immunoblotting

Lysates or IP eluates were resolved by 4–20% Criterion TGX Midi gels (Bio-Rad) and transferred to nitrocellulose membranes (GE) following the manufacturer’s protocol. Membranes were blocked in Intercept (TBS) blocking buffer (LI-COR) for 1 h at room temperature. Membranes were incubated overnight with primary antibodies at 4 °C and then with fluorophore-conjugated secondary antibodies (LI-COR) for 1 h at room temperature. All antibodies were used at the dilutions suggested by the manufacturers. The bands were visualized by LI-COR Odyssey Fc Imaging System. The images were processed with ImageJ.

### HEK *CTNNB1* Knockout with CRISPR-Cas9

Guide RNA (gRNA) targeting different human *CTNNB1* exons were designed with CHOPCHOP online tools^46^. The sequences of oligonucleotides were as follows: 5′-TGA GTA GCC ATT GTC CAC GC-3′ (exon 1), 5′-CTA ACA GCC GCT TTT CTG TC-3′ (exon 2), 5′-CTG TCT TTT CGC CGA CAA TC-3′ (nonsense), and 5′-CAA CAG TCT TAC CTG GAC TC-3′ (exon 3). Single guide RNA (sgRNA), Cas9 nuclease (HiFi) and duplex buffer were purchased from Integrated DNA Technologies. RNAs were reconstituted and diluted to 5 μM with duplex buffer; Cas9 protein to 10 μM with PBS. HEK cells were plated in 12-well plates at a seeding density of 1.0 × 10^5^ cells in 1 mL DMEM-complete a day before. For one reaction, sgRNA (20 μL), Cas9 (15 μL), DMEM (30 μL, no serum and antibiotics) and Lipofectamine RNAiMax (4 μL, Invitrogen) were mixed and incubated at room temperature for 20 min. The complex was treated to the cells in complete medium and incubated overnight at 37 °C and 5% CO_2_. The medium was changed and the cells were allowed to recover for 1 day. Cells were split to maintain 30–50% confluency. The sgRNA-Cas9 treatment was repeated 3 times. Cells were expanded and sorted with a flow cytometer (FACSMelody, BD) to 96-well plates to grow single cell colonies. The colonies were screened for low (< 5 ng/well at confluent) β-cat expression using a β-cat ELISA kit (Invitrogen). The selected colonies were verified with western blot. The knockout cell line was maintained in the same conditions as WT.

### Immunocytochemistry

Microscope cover glasses (12 mm, Fisher) were flamed and coated with 1 mL poly-D-lysine (0.1 mg/mL, Gibco) in a 12 well plate for 1 h at room temperature. HEK cells (WT, β-cat^KO^ or 1:4 mixture) were seeded at at a seeding density of 1.0 × 10^5^ cells and incubated for 3 days. Cells were fixed in 4% paraformaldehyde (PFA) in PBS for 30 min, quenched with 100 mM glycine, permeabilized with 0.3% Triton X-100, and blocked with 3% normal goat serum (MilliporeSigma). Primary (1:100, rabbit anti-β-cat, clone RM276, MilliporeSigma) and secondary (1:300, Alexa Fluor 568 goat anti-rabbit, MilliporeSigma) antibody incubations were performed at room temperature for 1 h, Hoechst (1:10,000, ThermoFisher) was incubated for 5 min, interspaced by multiple washes in PBS, and followed by mounting coverslips in ProLong Gold fixative (Life Technologies). Fixed cells were imaged on an Axioplan 2 microscope (Carl Zeiss) at room temperature using a × 40 objective lens. Captured images were processed in ImageJ.

## Supplementary Information

Supplementary Information 1.

Supplementary Information 2.

Supplementary Tables.

## Data Availability

The datasets analyzed during the current study are available in the PhosphoSitePlus (https://www.phosphosite.org/) and BioMuta (https://hive.biochemistry.gwu.edu/biomuta/) repositories. The data for β-catenin is available using Uniprot accession code P35222 in both databases. All data generated during this study are included in this published article (and its Supplementary Information files).

## References

[1] Tonks NK, Neel BG (2001). Combinatorial control of the specificity of protein tyrosine phosphatases. Curr. Opin. Cell Biol..

[2] Alonso A (2004). Protein tyrosine phosphatases in the human genome. Cell.

[3] Beck JR, Lawrence A, Tung AS, Harris EN, Stains CI (2016). Interrogating endogenous protein phosphatase activity with rationally designed chemosensors. ACS Chem. Biol..

[4] Hale AJ, Ter Steege E, den Hertog J (2017). Recent advances in understanding the role of protein-tyrosine phosphatases in development and disease. Dev. Biol..

[5] Hendriks WJ (2013). Protein tyrosine phosphatases in health and disease. FEBS J..

[6] Tonks NK (2006). Protein tyrosine phosphatases: From genes, to function, to disease. Nat. Rev. Mol. Cell Biol..

[7] Tonks NK (2013). Protein tyrosine phosphatases–from housekeeping enzymes to master regulators of signal transduction. FEBS J..

[8] Chan G, Kalaitzidis D, Neel BG (2008). The tyrosine phosphatase Shp2 (PTPN11) in cancer. Cancer Metastasis Rev..

[9] Loh ML (2004). Mutations in PTPN11 implicate the SHP-2 phosphatase in leukemogenesis. Blood.

[10] Tartaglia M (2003). Somatic mutations in PTPN11 in juvenile myelomonocytic leukemia, myelodysplastic syndromes and acute myeloid leukemia. Nat. Genet..

[11] Feldhammer M, Uetani N, Miranda-Saavedra D, Tremblay ML (2013). PTP1B: A simple enzyme for a complex world. Crit. Rev. Biochem. Mol. Biol..

[12] Casey GR, Stains CI (2018). Interrogating protein phosphatases with chemical activity probes. Chem. (Easton).

[13] Casey GR, Stains CI (2020). A fluorescent probe for monitoring PTP-PEST enzymatic activity. Analyst.

[14] Palma A (2017). Both intrinsic substrate preference and network context contribute to substrate selection of classical tyrosine phosphatases. J. Biol. Chem..

[15] Mrksich M (2008). Mass spectrometry of self-assembled monolayers: A new tool for molecular surface science. ACS Nano.

[16] Min DH, Su J, Mrksich M (2004). Profiling kinase activities by using a peptide chip and mass spectrometry. Angew. Chem. Int. Ed. Engl..

[17] Kuo HY, DeLuca TA, Miller WM, Mrksich M (2013). Profiling deacetylase activities in cell lysates with peptide arrays and SAMDI mass spectrometry. Anal. Chem..

[18] Wood SE (2017). A bottom-up proteomic approach to identify substrate specificity of outer-membrane protease OmpT. Angew. Chem. Int. Ed. Engl..

[19] Kightlinger W (2018). Design of glycosylation sites by rapid synthesis and analysis of glycosyltransferases. Nat. Chem. Biol..

[20] Szymczak LC, Huang CF, Berns EJ, Mrksich M (2018). Combining SAMDI mass spectrometry and peptide arrays to profile phosphatase activities. Methods Enzymol..

[21] Szymczak LC, Kuo HY, Mrksich M (2018). Peptide arrays: Development and application. Anal. Chem..

[22] Huang CF, Mrksich M (2019). Profiling protein tyrosine phosphatase specificity with self-assembled monolayers for matrix-assisted laser desorption/ionization mass spectrometry and peptide arrays. ACS Comb. Sci..

[23] Zhang ZY (1993). Substrate specificity of the protein tyrosine phosphatases. Proc. Natl. Acad. Sci. USA.

[24] Garaud M, Pei D (2007). Substrate profiling of protein tyrosine phosphatase PTP1B by screening a combinatorial peptide library. J. Am. Chem. Soc..

[25] Ren L (2011). Substrate specificity of protein tyrosine phosphatases 1B, RPTPalpha, SHP-1, and SHP-2. Biochemistry.

[26] Selner NG (2014). Diverse levels of sequence selectivity and catalytic efficiency of protein-tyrosine phosphatases. Biochemistry.

[27] Szymczak LC, Sykora DJ, Mrksich M (2020). Using peptide arrays to profile phosphatase activity in cell lysates. Chem. (Easton).

[28] Hornbeck PV (2015). PhosphoSitePlus, 2014: Mutations, PTMs and recalibrations. Nucleic Acids Res..

[29] Dingerdissen HM (2018). BioMuta and BioXpress: Mutation and expression knowledgebases for cancer biomarker discovery. Nucleic Acids Res..

[30] Tucci V (2014). Dominant beta-catenin mutations cause intellectual disability with recognizable syndromic features. J. Clin. Invest..

[31] Kan Z (2010). Diverse somatic mutation patterns and pathway alterations in human cancers. Nature.

[32] Jho EH (2002). Wnt/beta-catenin/Tcf signaling induces the transcription of Axin2, a negative regulator of the signaling pathway. Mol. Cell. Biol..

[33] van Veelen W (2011). beta-catenin tyrosine 654 phosphorylation increases Wnt signalling and intestinal tumorigenesis. Gut.

[34] McEwen AE, Escobar DE, Gottardi CJ (2012). Signaling from the adherens junction. Subcell. Biochem..

[35] Daugherty RL, Gottardi CJ (2007). Phospho-regulation of Beta-catenin adhesion and signaling functions. Physiol. (Bethesda).

[36] Roura S, Miravet S, Piedra J, Garcia-de-Herreros A, Dunach M (1999). Regulation of E-cadherin/Catenin association by tyrosine phosphorylation. J. Biol. Chem..

[37] Roper JC (2018). The major beta-catenin/E-cadherin junctional binding site is a primary molecular mechano-transductor of differentiation in vivo. Elife.

[38] Yan HX (2006). Protein-tyrosine phosphatase PCP-2 inhibits beta-catenin signaling and increases E-cadherin-dependent cell adhesion. J. Biol. Chem..

[39] Simoneau M, Coulombe G, Vandal G, Vezina A, Rivard N (2011). SHP-1 inhibits beta-catenin function by inducing its degradation and interfering with its association with TATA-binding protein. Cell. Signal..

[40] Winer IS, Bommer GT, Gonik N, Fearon ER (2006). Lysine residues Lys-19 and Lys-49 of beta-catenin regulate its levels and function in T cell factor transcriptional activation and neoplastic transformation. J. Biol. Chem..

[41] Tinti M (2012). The 4G10, pY20 and p-TYR-100 antibody specificity: Profiling by peptide microarrays. New Biotechnol..

[42] Aleshin A, Finn RS (2010). SRC: A century of science brought to the clinic. Neoplasia.

[43] Varone A, Spano D, Corda D (2020). Shp1 in solid cancers and their therapy. Front. Oncol..

[44] Takai A, Mieskes G (1991). Inhibitory effect of okadaic acid on the p-nitrophenyl phosphate phosphatase activity of protein phosphatases. Biochem. J.

[45] Welte S (2005). 6,8-Difluoro-4-methylumbiliferyl phosphate: A fluorogenic substrate for protein tyrosine phosphatases. Anal. Biochem..

[46] Labun K (2019). CHOPCHOP v3: Expanding the CRISPR web toolbox beyond genome editing. Nucleic Acids Res..

