# Supplementary material for: Tyrosine phosphatase activity is restricted by basic charge substituting mutation of substrates
Source: Sci Rep. 2022 Sep 5;12:15095. doi: 10.1038/s41598-022-19133-4 (PMC9445012; doi:10.1038/s41598-022-19133-4)
Supplement: Supplementary file 1 — Supplementary Information 1. [file 41598_2022_19133_MOESM1_ESM.docx]

**ASSOCIATED CONTENT**

**Supporting Information.** Characterization of HEK β-cat^KO^ cells, cancer database search results.
