# Supplementary material for: Tyrosine phosphatase activity is restricted by basic charge substituting mutation of substrates
Source: Sci Rep. 2022 Sep 5;12:15095. doi: 10.1038/s41598-022-19133-4 (PMC9445012; doi:10.1038/s41598-022-19133-4)
Supplement: Supplementary file 2 — Supplementary Information 2. [file 41598_2022_19133_MOESM2_ESM.docx]

**Supporting Information**

**Tyrosine Phosphatase Activity Is Restricted by Basic Charge Substituting Mutation of Substrates**

Che-Fan Huang^1^, Cara J. Gottardi^2,3^*, and Milan Mrksich^1,4,5^*

^1^Department of Chemistry, Northwestern University, Evanston, IL 60208, USA

^2^Division of Pulmonary and Critical Care, Department of Medicine, Northwestern University, Chicago, IL 60611, USA

^3^Biochemistry and Molecular Genetics, Northwestern University, Chicago, IL 60611, USA

^4^Department of Biomedical Engineering, Northwestern University, Evanston, IL 60208, USA

^5^Department of Cell & Developmental Biology, Northwestern University, Chicago, Illinois 60611, USA

*Corresponding authors.

Cara J. Gottardi. Email: c-gottardi@northwestern.edu

Milan Mrksich. E-mail: milan.mrksich@northwestern.edu.

**Table of Contents**

| Creating β-cat^KO^ HEK 293T cell lines using CRISPR-Cas9 | 2-3 |
| --- | --- |
| Fig. S1. Creating β-cat^KO^ HEK 293T cell lines and their characterization | 3 |
| Full-length western blots  References | 4-8  9 |
| Table S1. Basic mutations at -1 | x |
| Table S2. Basic mutations at +1 | x |

**Creating β-cat^KO^ HEK 293T cell lines using CRISPR-Cas9.** Guide RNAs targeting 3 different human *CTNNB1* exons (guide 1, 2, 4, Fig. S1a) as well as a nonsense sequence (guide 3, reverse of guide 2) were designed using CHOPCHOP online tools.^1^ Wildtype HEK 293T cells were transfected with each sgRNA-Cas9 complex overnight 4 times and the bulk cells were expanded and blotted for β-cat (Fig. S1b). Guide 2 and 4 showed about 50% knockout in bulk cells whereas those treated with exon 1 and the nonsense guides had β-cat expressed like WT. The result was consistent with previously reported efforts creating β-cat^KO^ cell using the same gRNA sequences.^2^ Cells treated with gRNA 2 and 4 were then sorted to 96-wells plates (1 cell/well) with a flow-cytometer, expanded and primarily screened with ELISA for low-β-cat expressing colonies. These colonies were further expanded and blotted for β-cat (Fig. S1c). Several colonies from both guides showed a complete knockout on western blots (rabbit anti-β-cat, clone RM276). We also observed that clone 16 and 18 had a more significant upregulation of plakoglobin (γCat), which was previously identified to rescue the junction in β-cat^KO^ cells.^3^

We next used an AXIN2 antibody to verify the β-cat^KO^ cells. To avoid species overlap, we switched to a mouse β-cat antibody (BD). To our surprise, some clones previously appeared to be β-cat null (anti-β-cat-RM276) showed a fragment below 75 kD using the BD antibody (Fig. S1d). This was most likely a c-terminus fragment as the antibody was generated with the immunogen β-cat a.a. 571-781. The fragment was also active to turn on Wnt pathway and induce AXIN2 expression with or without the Wnt pathway agonist and GSK3 inhibitor, LiCl.^4^ We identified that clone 16 was a complete β-cat^KO^ cell line without c-terminus fragments and background AXIN2 expression (Fig. S1e). The cell line was further verified for knock by immunocytochemistry. A separate culture of WT and KO cells and staining with a β-cat antibody showed a distinct difference where β-cat was observed at the junctions in WT but not in KO (Fig. S1f, first two images). A co-culture of WT and KO cells (Fig. S1f, last image) clearly demonstrated on the same slide that β-cat only distributed at the junctions in WT (upper right area) but not KO (lower left). HEK 293T β-cat^KO^ clone 16 was used for the further studies in the paper.


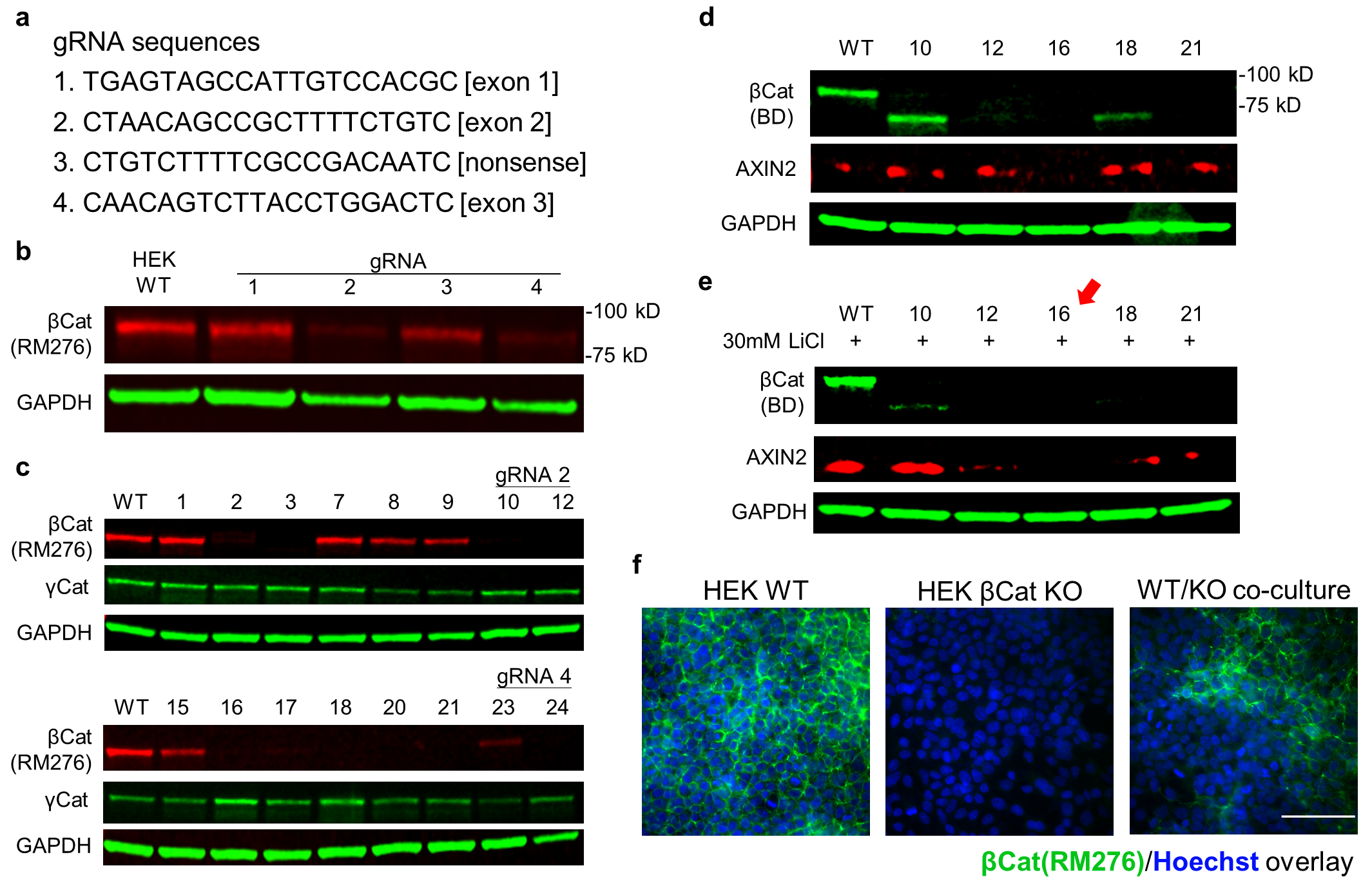


**Fig. S1. Creating β-cat^KO^ HEK 293T cell lines and their characterization. a**, gRNA sequences used for CRISPR-Cas9 β-cat knockout. **b**, Bulk cells blotted for β-cat after sgRNA-Cas9 complex treatments. **c**, Validation of knockout by single cell colonies. **d**, Validation using a c-terminus β-cat and AXIN2 antibodies. **e**, Screening for AXIN2-null cells with LiCl (30 mM) treatment. **f**, Separate and co-culture of WT and β-cat ^KO^ HEK 293T cells. Scale bar: 50 μm.

**FULL-LENGTH WESTERN BLOTS**

**
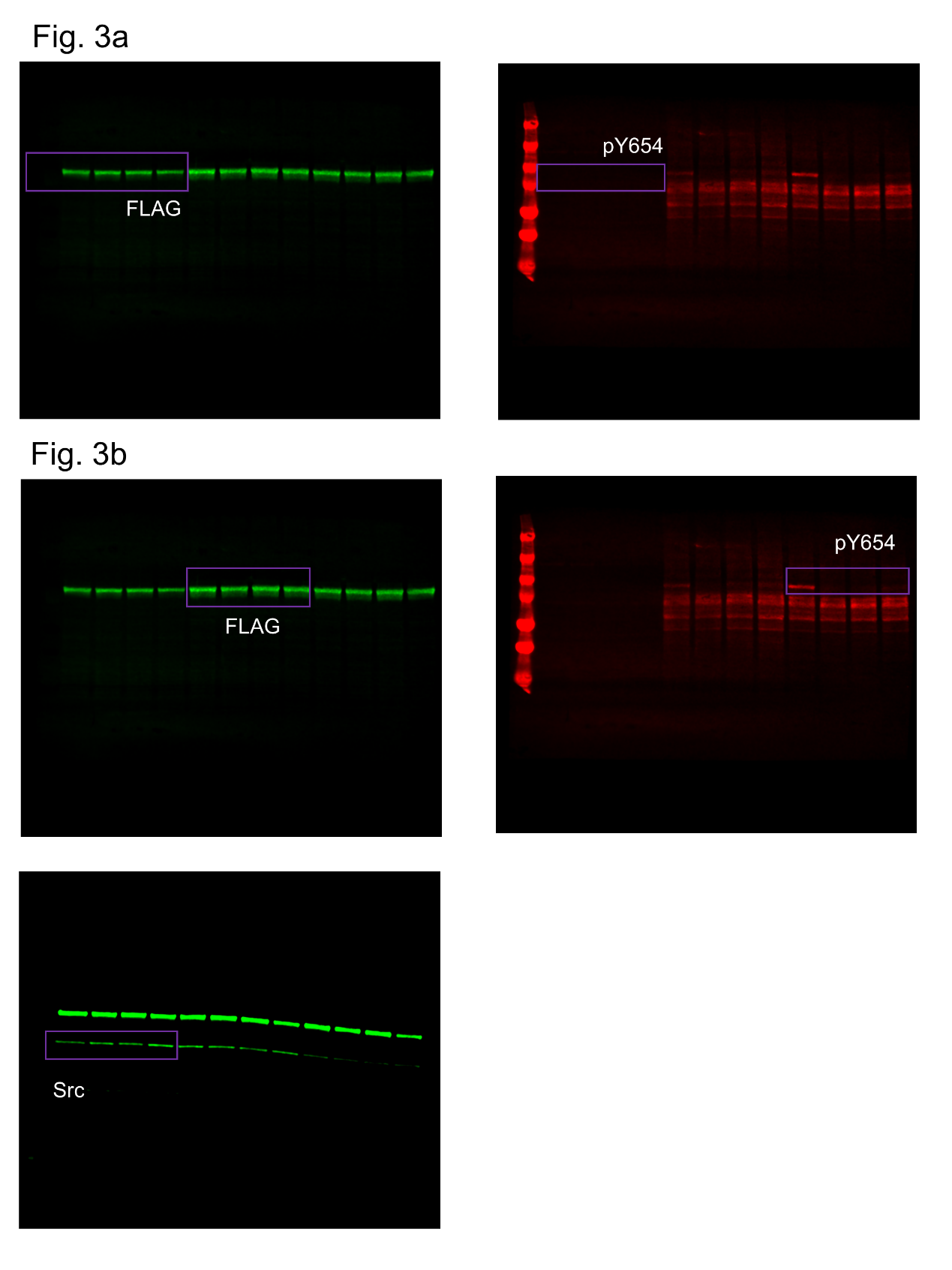

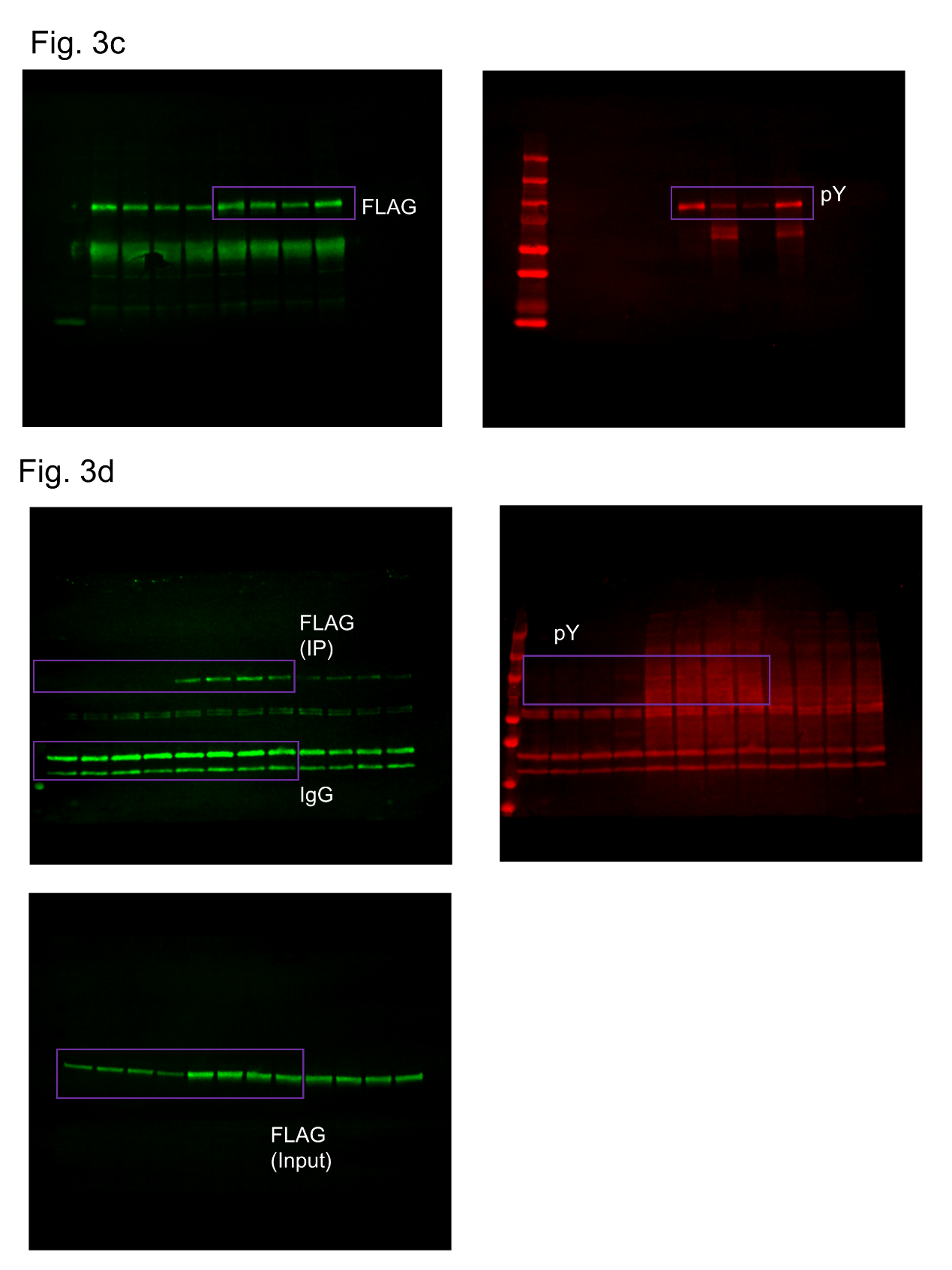

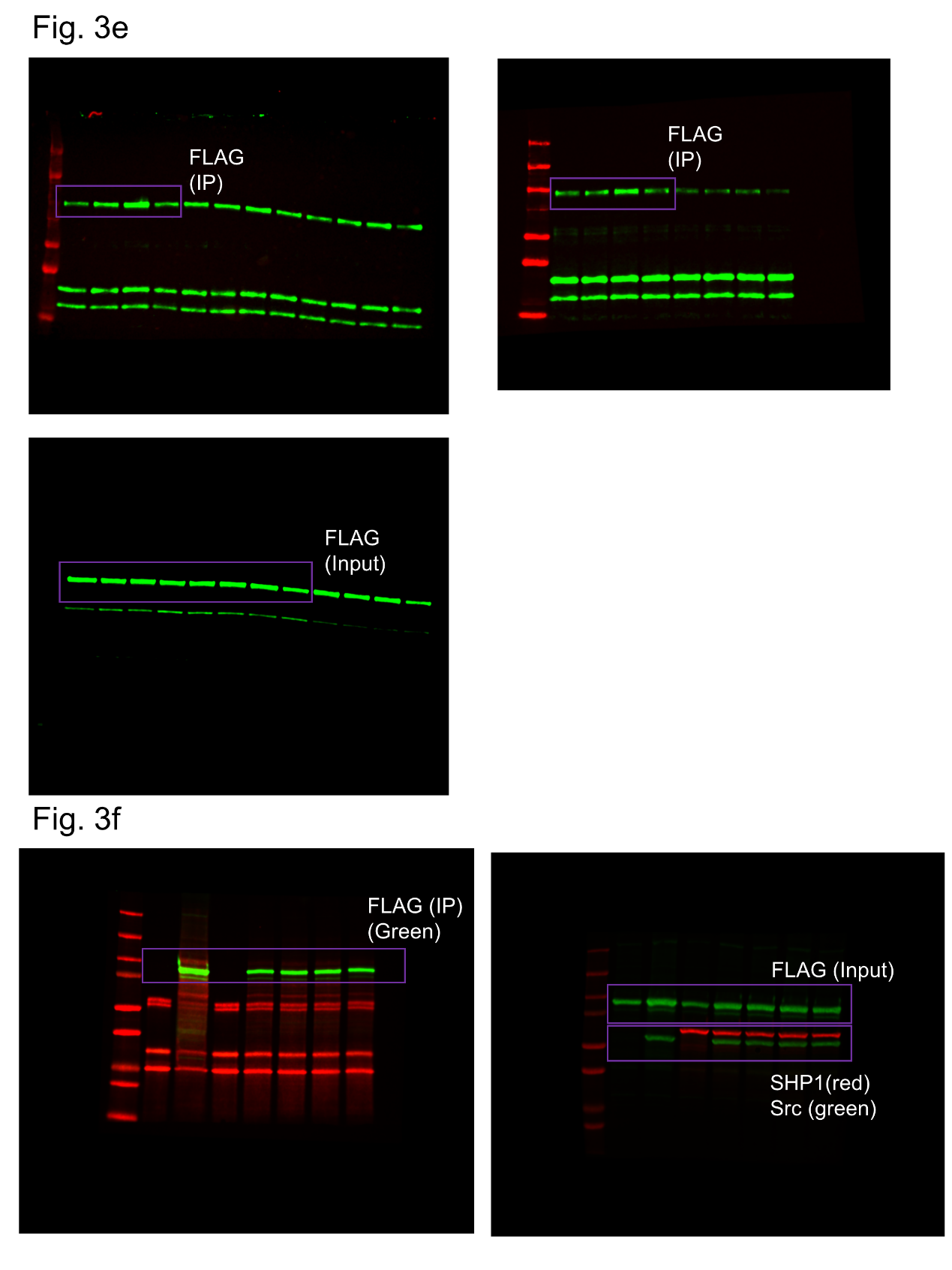

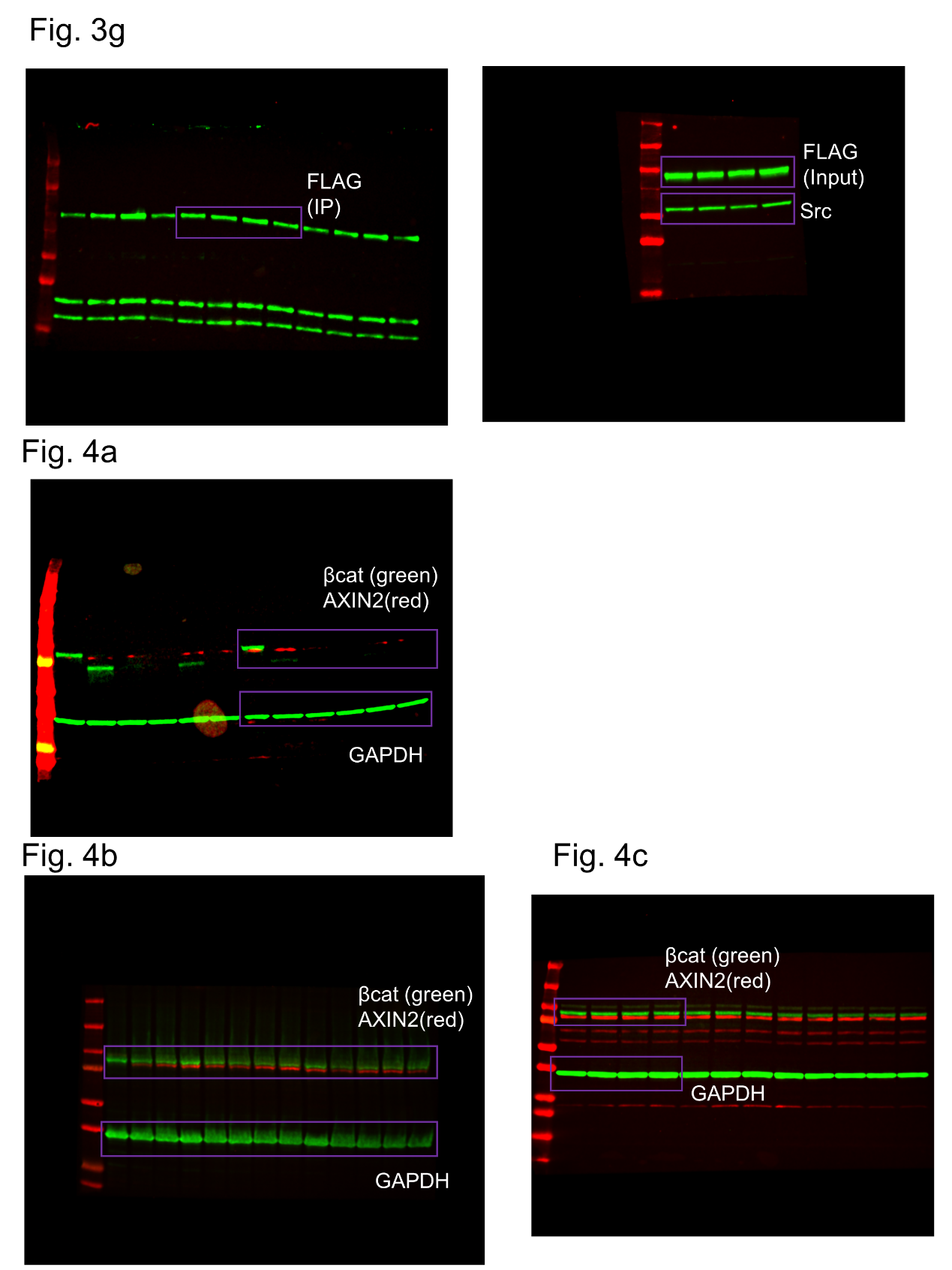

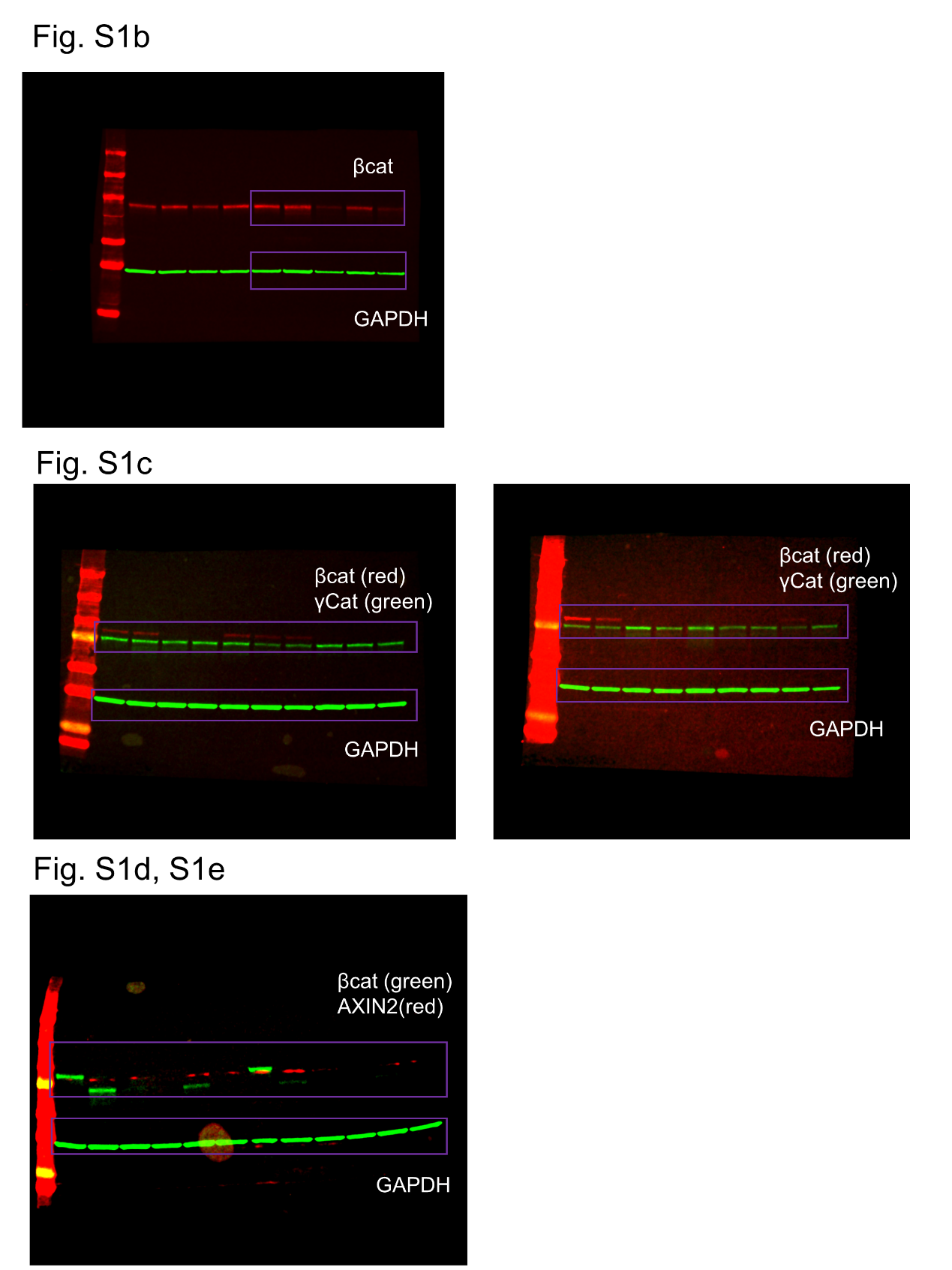
**

**REFERENCES**

1 Labun, K. *et al.* CHOPCHOP v3: expanding the CRISPR web toolbox beyond genome editing. *Nucleic Acids Res.* **47**, W171-W174, doi:10.1093/nar/gkz365 (2019).

2 Guan, L. *et al.* Knockout of CTNNB1 by CRISPR-Cas9 technology inhibits cell proliferation through the Wnt/beta-catenin signaling pathway. *Biotechnol. Lett* **40**, 501-508, doi:10.1007/s10529-017-2491-2 (2018).

3 Kobayashi, W. & Ozawa, M. The epithelial-mesenchymal transition induced by transcription factor LEF-1 is independent of beta-catenin. *Biochem Biophys Rep* **15**, 13-18, doi:10.1016/j.bbrep.2018.06.003 (2018).

4 Jho, E. H. *et al.* Wnt/beta-catenin/Tcf signaling induces the transcription of Axin2, a negative regulator of the signaling pathway. *Mol. Cell. Biol.* **22**, 1172-1183, doi:10.1128/mcb.22.4.1172-1183.2002 (2002).
